# Secure aggregation for heterogeneous enterprise data based on federated meta-learning

**DOI:** 10.1038/s41598-025-34735-4

**Published:** 2026-01-26

**Authors:** Chun Yang, Yining Ma

**Affiliations:** Energy Development Research Institute, China Southern Power Grid, Guangzhou, 510663 China

**Keywords:** Federated meta-learning, Data aggregation, Data collection, Performance evaluation, Engineering, Mathematics and computing

## Abstract

This paper proposes a secure and efficient data aggregation approach for heterogeneous enterprise data, leveraging federated meta-learning (FML) and data consolidation. The proposed approach addresses critical challenges in enterprise data, including data privacy, heterogeneous data distributions, and communication constraints. Specifically, the proposed approach enables decentralized devices to collaboratively train local models, which are aggregated by the server into a global model using meta-learning. Then, a consortium blockchain is used to ensure secure, immutable storage of aggregated data through a dual-chain structure: lightweight fluffy chains for temporary storage and heavyweight bulky chains for permanent records. In further, the proposed approach incorporates robust security mechanisms, such as XF authentication, timestamp-based counters to thwart replay attacks, asymmetric encryption for secure key exchange, and a Hampel filter to detect and mitigate model poisoning. Simulations results under Secure Water Treatment (SWaT) dataset are finally provided to demonstrate the superiority of the proposed approach. Specifically, the proposed approach achieves a lower validation delay scaling efficiently with training history size over the competing ones. Additionally, the proposed approach can enhance the system security by increasing the attack failure probability up to 15% over the competing ones.

## Introduction

### Research background

Industrial Internet of Things (IIoT) has emerged as a transformative paradigm in modern manufacturing and industrial operations, enabling the seamless connection of machines, sensors, controllers, and systems across complex, distributed environments^1,2^. Through IIoT, massive volumes of data are generated at the edge, from factory floors, production lines, and operational equipment, requiring robust infrastructures to support real-time data transmission, processing, and analysis. These environments are inherently heterogeneous, involving diverse hardware, communication protocols, and software platforms, which complicates system interoperability and data unification^3–5^. As IIoT deployments scale across geographies and organizational boundaries, the challenge of managing distributed and disjointed data streams becomes increasingly critical. In this context, enterprise data-often considered the backbone of business intelligence and decision-making should be integrated with IIoT-generated data despite fundamental differences in structure, semantics, and governance. Enterprise data systems, which traditionally reside in centralized databases or ERP platforms, are now required to interact with dynamic, real-time data streams from highly varied IIoT sources^6,7^. The heterogeneity of these data sources necessitates semantic models, ontologies, and middleware solutions to bridge the gap between operational technology and information technology. Moreover, the distributed nature of IIoT implies that enterprise data is no longer static or isolated; rather, it is part of a fluid and interconnected digital ecosystem. To manage this complexity, some techniques such as cloud-edge collaboration, data-as-a-product architectures, and decentralized identity management have been proposed, with the aim to ensure that enterprise data can be accessed, analyzed, and trusted across heterogeneous and distributed industrial environments^8–10^.

### Related works

To tackle the data complexity in IIoT environments, federated learning (FL) has been adopted as a privacy-preserving machine learning paradigm in which model training is collaboratively performed across decentralized clients without requiring raw data to be shared^11,12^. By allowing local data to remain on individual devices or silos, FL enables the data privacy and regulatory compliance while still benefiting from collective intelligence. This framework has been implemented in diverse domains such as mobile devices, healthcare systems, and IoT environments, where sensitive data is generated in a highly distributed manner^13,14^. However, significant performance challenges have been introduced due to the nature of data distribution across clients. In many real-world scenarios, the data held by each client is non-independent and identically distributed (non-IID), resulting in heterogeneity that may severely deteriorate the model convergence and generalization. This data heterogeneity, arising from differences in feature distributions, label distributions, and data volume, has been shown to cause divergence among local updates, leading to slow convergence, biased models, and even instability in the learning process^15,16^. Moreover, traditional aggregation algorithms such as federated averaging (FedAvg) have been found to be suboptimal in handling statistical heterogeneity, often requiring personalization strategies or adaptive weighting schemes to balance contributions from disparate clients. To address these issues, some techniques such as clustered federated learning, adaptive optimization methods, and proximal regularization have been proposed to mitigate the effect of heterogeneous data distribution while maintaining the efficiency and scalability of the federated learning^17,18^. Nevertheless, achieving a robust and fair model performance across non-uniformly distributed datasets remains a research challenge in the federated learning.

Besides the data complexity, data privacy has also attracted much attention from researchers, particularly on distributed systems like cloud-enabled healthcare and the Internet of medical Things (IoMT)^19^. In this field, some cryptographic methods have been proposed to protect the data privacy, such as the spectrasafe encryption and dynamic *k*-anonymity algorithm for cloud privacy^20^, information entropy-based fully homomorphic encryption combined with RSA for healthcare data^21^, and the contextual polynomial-based data protection model for clinical data^22,23^. Moreover, the blockchain technology has been proposed to safeguard the distributed data and transactions^24–26^, which provides a platform for building secure and trusted frameworks, exemplified by the use of a consortium Blockchain structure in FinTech for scalable customer data protection^25^. Additionally, secure key agreement protocols with trust evaluation have been investigated in financial contexts like rotating savings and credit associations^24^, and a trusted system was established for managing financial contributions with creditworthiness evaluation^26^. Besides, the authors in^23^ proposed a novel SC-D$\ell$DA algorithm, to safeguard the data privacy in cloud-enabled multi party computation settings.

Meta-learning, often referred to as “learning to learn”, has been explored extensively in recent years, to enable models to rapidly adapt to new tasks with minimal data or computational effort^27,28^. In conventional machine learning, models are trained from scratch for each task, requiring significant amounts of data and time. In contrast, meta-learning frameworks are structured to extract transferable knowledge across multiple learning episodes, thereby allowing quick generalization to unseen tasks^29,30^. This has been particularly useful in few-shot and zero-shot learning settings, where traditional deep learning models typically struggle due to data scarcity^31^. Multiple paradigms have been investigated within the meta-learning domain, including model-based, metric-based, and optimization-based approaches^32–34^. In these frameworks, learning rules, initialization parameters, or even neural architectures are adjusted based on meta-objectives defined over task distributions rather than single-task losses. More recently, meta-learning has been integrated with deep learning to enhance its flexibility, generalization, and sample efficiency^35^. Deep neural networks have been employed as base learners or meta-learners, enabling hierarchical abstraction and multi-level learning. Additionally, adaptive optimizers and ensemble strategies have been meta-trained to dynamically guide deep learning processes, especially in environments characterized by data variability or limited annotations^36^. As a result, meta-learning has been adopted as a powerful augmentation strategy for deep learning systems, particularly in applications involving dynamic, non-stationary, or resource-constrained settings.

**Table 1:** Comparison of state-of-the-art and proposed scheme.

Scheme	FL	ML	Blockchain	Heterogeneous data	Dedicated anti-attack
FL techniques^17,18^	$\checkmark$	$\times$	$\times$	Partial	$\times$
Cryptographic methods^20,21^	$\times$	$\times$	$\times$	No	$\checkmark$
Blockchain frameworks^25,26^	Partial	$\times$	$\checkmark$	No	$\checkmark$
General ML/DL^29,30^	$\times$	$\checkmark$	$\times$	Partial	$\times$
Proposed scheme	$\checkmark$	$\checkmark$	$\checkmark$	$\checkmark$	$\checkmark$

### Motivation

To summarize the existing literature, while FL effectively addresses the data privacy, it still faces the significant challenge coming from the non-IID data with statistical heterogeneity, which deteriorates the model convergence and generalization. Moreover, although cryptographic and Blockchain techniques have been applied to enhance the data privacy and integrity, there is a lack of integrated solutions that leverage federated meta-learning (FML) to tackle the key non-IID problem while simultaneously establishing a robust security mechanism against complex threats like model poisoning and replay attacks in enterprise environments. Specifically, a comprehensive architecture combining the generalization of FML with the immutability of a Blockchain and dedicated attack mitigation strategies remains underexplored. A detailed comparison of key existing works and their differences from our proposed scheme is provided in Table 1. Motivated by these research gaps, we will investigate a secure and efficient data aggregation based on FML and a dual-chain blockchain structure in this paper.

### Contributions

This paper presents a secure and efficient data aggregation framework for handling heterogeneous enterprise data by integrating federated meta-learning with data consolidation. The proposed system is designed to address several pressing challenges in enterprise environments, including data privacy, statistical heterogeneity across distributed sources, and limited communication capabilities. In this architecture, decentralized edge devices collaboratively train local models, which are then aggregated by the server into a global model through meta-learning to enhance the generalization across non-IID data. To ensure data integrity and verifiability, a consortium blockchain is employed, featuring a dual-chain structure comprising lightweight “fluffy chains” for transient storage and heavyweight “bulky chains” for permanent archival. The system security is further reinforced through a comprehensive set of mechanisms: XF-based mutual authentication, timestamp-based counters to prevent replay attacks, asymmetric cryptography for secure key exchange, and a Hampel filter for detecting and mitigating model poisoning attempts. Simulation results are finally provided on under Secure Water Treatment (SWaT) dataset to validate the effectiveness of the proposed approach. In particular, the proposed approach achieves a lower blockchain validation latency with the accumulated training history over the competing ones. Additionally, the proposed approach significantly boosts the system security by increasing the attack failure probability by up to 15% over the competing ones.

The remainder of this paper is organized as follows. After the introduction in “Introduction”, “System model” describes the overall system model of federated learning on heterogeneous enterprise data. Then, “Proposed secure data consolidation process” details the proposed secure data consolidation process and its security analysis, followed by the computational complexity analysis. After that, “Simulations results and discussions” presents extensive simulation results and discussions, and finally, “Conclusions” concludes the whole paper and outlines possible directions for future research. The main notations used in this paper is summarized in Table 2.

**Table 2:** List of main notations.

Symbol	Explanation
$\tilde{Q\ }$	Consolidator/server coordinating collaborators and aggregation
$\tilde{N\ }$	Collaborator/client device participating in training
$\tilde{A\ }$	MEC server used for offloading/committing records to blockchain
$\sigma$	Public key
$\beta$	Private key
*nd*	Confidential token used in secure messaging
$\nu ,\nu _n$	Temporal nonce broadcast/generated by collaborator
$\Phi$	Symmetric session key for encrypting model updates/parameters
XF	XOR-filter-based identity authentication
*qdj*	XF dataset/filter constructed from enrolled public keys
$P_l(\cdot )$	Hash functions ($l\in \{0,1,2\}$) used in XF
Hampel filter	Robust outlier filtering for poisoned/abnormal updates
$\phi$	Global/meta model parameters
$\phi _n$	Local parameters for collaborator *n*
$\nabla \phi _n$	Reported model update/gradient from collaborator *n*
$K_n(\cdot )$	Loss function for client *n*
*i*MAML	Meta-learning with implicit gradients used in the scheme
$\rho _n$	Local dataset at collaborator *n*
$\rho _n^g,\rho _n^r$	Support set/query set for task adaptation and validation
$H_n$	Number of meta-tasks at client *n*
$\Psi$	Regularization coefficient in the inner-level objective
FedAvg	Aggregation rule for computing $\phi$ from $\{\phi _n\}$
$N, v_k$	Total collaborators/active contributors per round
*W*	Number of trainable parameters (model size)
$G, T_{cg}$	Inner-loop steps/implicit-gradient iteration number

## System model

**Fig. 1:**
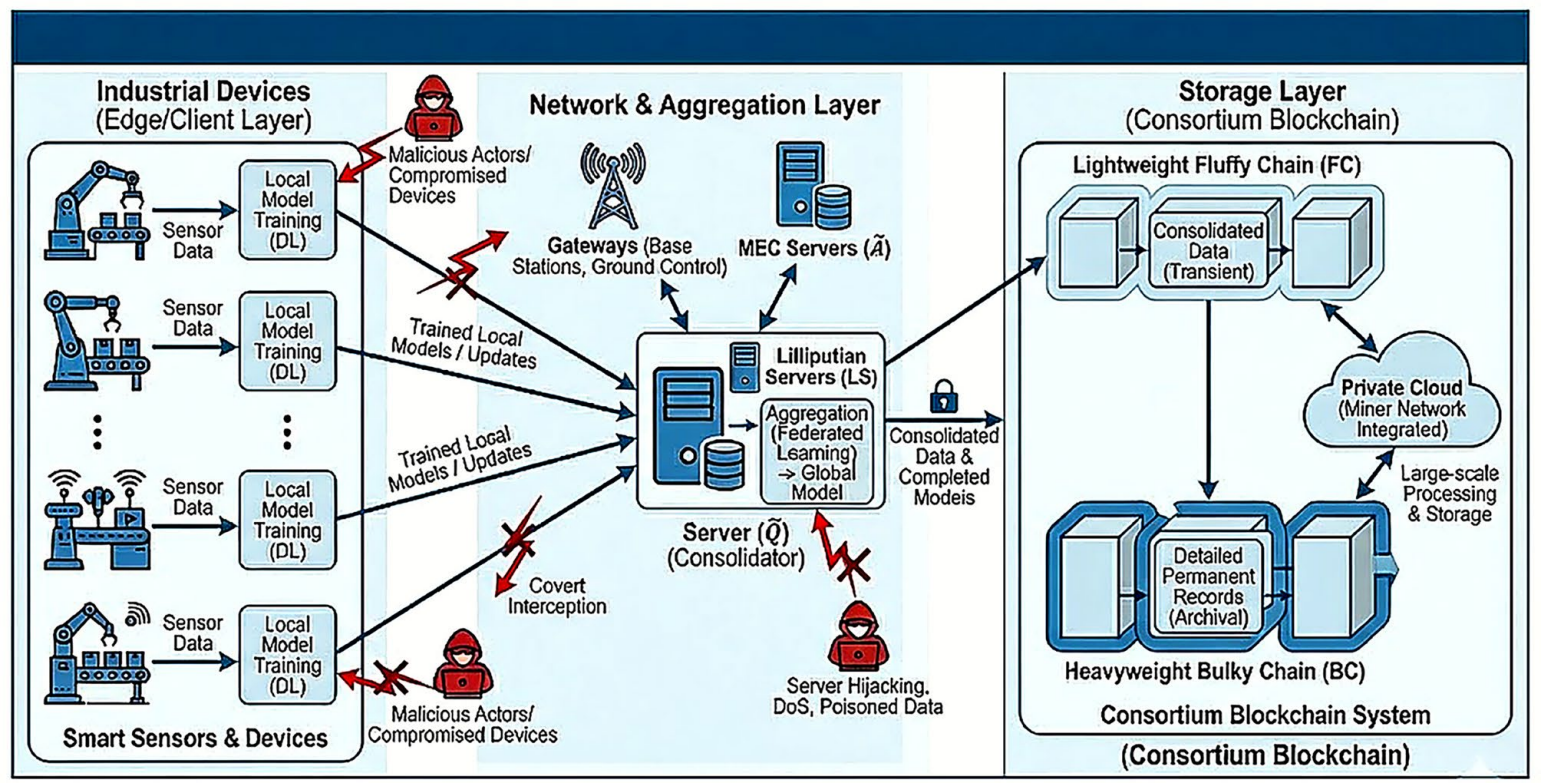
System model of distributed federated learning on heterogeneous enterprise data, with a split blockchain structure.

In this paper, we consider a distributed federated learning system on heterogeneous enterprise data, which is collected as trained models via a server and securely stored using a split blockchain structure, shown in Fig. 1. In this system, multiple industrial devices equipped with various smart sensors are deployed in industrial settings, responsible for training with local data. The federated learning involves deep learning (DL) models, which include both global and local models. In particular, local models are trained using sensor data, while global models are generated through local model aggregation. The server acts as consolidators to train and store data upon task completion. A consortium blockchain system is implemented to ensure the data storage, using a dual-chain structure, where lightweight fluffy chains (FC) is for consolidated data while heavyweight bulky chains (BC) is for detailed permanent records. Gateways, including base stations and ground control facilities, are responsible for managing the data, ensuring efficient processing and transmission. The system’s server infrastructure includes various types, such as “lilliputian servers (LS)”, which leverage dew computing to the missions in both offline and online scenarios. Additionally, mobile edge computing (MEC) servers are positioned at gateways to provide real-time assistance to the server. A private cloud integrated within the miner network further enhances the system’s capabilities for large-scale data processing and storage.

In the considered system, the server facilitate the data consolidation by training models using sensor data from industrial devices. Once the training is complete, the models are stored securely in a blockchain. However, this process introduces security vulnerabilities. For instance, the malicious actors, such as compromised industrial devices, may send incorrect update to manipulate and corrupt the global model. Attackers could attempt to hijack the server to steal data or disrupt the training process. Additionally, unauthorized network access might enable covert interception of communication between the devices and server. Moreover, attackers could launch denial-of-service attacks by flooding the system with outdated data or injecting poisoned data into the server storage, posing significant security risks.

## Proposed secure data consolidation process

### Preliminary preparations

In the considered system, all entities should undergo a registration process. Each entity generates a private key, denoted as $\beta = \mathcal {B}((ac, dg, dd))$, where $\mathcal {B} : \{0,1\}^* \mapsto \{0,1\}^{bk}$. This private key is derived using the MAC address $ac$, the current timestamp $dg$, and a random input text $dd$. A corresponding public key is then derived from the private key, represented as $\sigma = (\mathcal {M}_j, \mathcal {M}_f) \times \beta$, where $\mathcal {M}$ represents an elliptic curve point. Once the keys are generated, the entity transmits $\sigma$ along with other basic information to the nearest MEC server to complete the registration. Within the network, $\sigma$ acts as the entity’s identity. Upon receiving the registration request, the MEC server $\tilde{A}$ generates a confidential token, denoted as $nd= \mathcal {P}((dg, kn, qs, \dots ))$, where $\mathcal {P} : \{0,1\}^* \mapsto \{0,1\}^{bk}$. This token is derived from the provided basic information, which includes the current timestamp $dg$, location $kn$, device details $qs$, and other relevant data. The MEC server $\tilde{A}$ then stores this information in the blockchain, denoted as $\overline{un} = \bigcup (nd, \sigma , kn, \dots )$. Finally, $\tilde{A}$ returns the confidential token $nd$ to the sender, which is essential for establishing a secure communication channel between entities.

Before deploying the server, a series of preparatory steps is required to ensure secure execution of the training process. The considered system integrates an XF authentication mechanism in its security design. To implement XF authentication, a dataset $qd^j$ is prepared, where each entity’s public key serves as its identity. To generate an entity dataset, a table $\bar{du}$ of length $dk= 1.23 \times | {vd} | + 32$ is created, where ${vd} = \bigcup (\sigma _l)$ represents the collection of entity public keys involved in training, and $s$ denotes the dataset length.

In the XF process, three different hash functions are used, denoted as $\mathcal {P}_l: \{0,1\}^* \mapsto f| f\in \left[ \frac{dk\times l}{3}, \frac{dk\times (l+1)}{3} \right]$, $\forall l\in [0,2] \wedge l\in \mathbb {S}$, which determine the placement of items in the dataset. Each entity in ${vd}$ is hashed three times, and the generated hashes are appended to the corresponding sets. The XF process searches for locations containing single keys and forms a stack $\bar{g}$ defined as $\bar{g} = \bigcup (\{vd, l\} | | \bar{du}_l| = 1, \forall l\in [0, |\bar{du}|] )$. During the selection, XF removes $vd$ from the three hashed locations. Once this step is completed, $qd^j$ is created by performing an XOR operation on $vd$ using hash functions, given by,

1 $$\begin{aligned} qd^j_{wl} = \mathcal {O}(w_{vd}) \oplus \left( \bigoplus _{\forall b\in [0,2]} qd^j_{\mathcal {P}_b(w_{vd})} \right) . \end{aligned}$$

Then, a multilayer perceptron (MLP) model, denoted as $a$, is constructed for training. To support industrial devices, $a$ is designed to be lightweight and consists of multiple blocks, each containing a dense layer $qk$, batch normalization $us$, an activation function $co$, and a dropout layer $q$. In each block, the input $j$ is first passed through a dense layer,

2 $$\begin{aligned} y^k_b= \sum _{\forall l\in [0, B_{k-1}]} \alpha ^k_{b,l} \times j^{k-1}_l+ u^k_b, \forall k\in [0, K), \forall b\in [0, B_k), \end{aligned}$$

where $\alpha$ represents weights, $u$ is the bias, $k$ represents the layer, $b$ is the neuron index, and $l$ refers to the neuron in the previous layer. After that, the batch normalization is applied,

3 $$\begin{aligned} y_l= \frac{j_l- \omega }{\sqrt{\theta + \eta }} \times \iota + \gamma , \forall l\in [0, r_k], \end{aligned}$$

with

4 $$\begin{aligned} \omega = \frac{1}{r_k} \times \sum _{\forall l\in [0, r_k]} j_l, \quad \theta = \frac{1}{r_k} \times \sum _{\forall l\in [0, r_k]} (j_l- \omega )^2. \end{aligned}$$

Here, $\iota$ and $\gamma$ are learnable parameters, $\omega$ represents the mean, and $\theta$ denotes the variance. The activation function applied is parametric rectified linear unit (PReLU). To mitigate the overfitting, a dropout layer is incorporated, following the Bernoulli distribution. In this work, it is assumed that the consolidator (i.e., the server $\tilde{Q}$) operates in a non-line-of-sight (NLOS) environment. As a result, continuous connectivity with the blockchain’s main node $un$ is not always available. To address this, a lightweight storage layer is introduced, which allows $\tilde{Q}$ to store data locally until connectivity with BC is restored. A genesis block $u_{FC}$ is created for FC, storing $\langle nd, a, mission\_information, \sigma _{\tilde{A}} \rangle$. A reference block $un^d_{FC}$ is later added to BC, shown as $u^d_{BC} = \langle un^d_{FC}, a, \sigma _{\tilde{Q}}, training\_history \rangle$. A standard dataset $qd^j_g$ is then prepared to evaluate the performance of $a$ during the training. Once all preparatory steps are completed, the model $a$ and its associated modules are deployed into $\tilde{Q}$.

### Pre-training provisions

When the server $\tilde{Q}$ reaches its destination, it broadcasts beacon messages $ua$ to invite devices as collaborators, represented as,

5 $$\begin{aligned} ua= \langle \tilde{Q}, \nu \rangle , \end{aligned}$$

where $\nu$ is a temporal nonce. Upon receiving $ua$, a collaborator $\tilde{N}$ transmits its public key and an encrypted message using $nd$, along with its temporal nonce $\nu _n$. The entire message is then encrypted using $\sigma _{\tilde{Q}}$ before being sent back to $\tilde{Q}$,

6 $$\begin{aligned} va= \mathcal {V}_{\sigma _{\tilde{Q}}} (\langle \mathcal {V}''_{nd}(\sigma _{\tilde{N}}), \sigma _{\tilde{N}}, \nu _n\rangle ). \end{aligned}$$

Upon receiving $va$, $\tilde{Q}$ decrypts it using the decryption function,

7 $$\begin{aligned} qa= \mathcal {Q}_{\beta _{\tilde{Q}}} (va). \end{aligned}$$

After decryption, $\tilde{Q}$ verifies the sender’s authenticity using XF authentication. This involves generating a fingerprint and three hash values using $\sigma _{\tilde{N}}$, then performing an XOR operation to compare them for consistency,

8 $$\begin{aligned} t= {\left\{ \begin{array}{ll} \text {True,} & \text {If } \mathcal {O}(\sigma _{\tilde{N}}) = \left( \bigoplus _{\forall z\in [0,2]} qd^j_{\mathcal {P}_z(\sigma _{\tilde{N}})} \right) ,\\ \text {False,} & \text {Otherwise}. \end{array}\right. } \end{aligned}$$

Finally, $\tilde{Q}$ conducts a CNA process by applying,

9 $$\begin{aligned} t= t\wedge (\nu _{n,v} > \nu _{n,v-1}). \end{aligned}$$

If the request is invalid, $\tilde{Q}$ maintains two lists: a vulnerable list for potentially untrusted collaborators, and a malicious list for confirmed attackers. After two invalid attempts, the transmitter is added to the vulnerable lis*. If invalid attempts continue beyond a defined threshold $l_c$, the transmitter is moved to the malicious list. For valid transmitters, $\tilde{Q}$ sends a final acknowledgment $cb$, encrypted as,

10 $$\begin{aligned} va= \mathcal {V}_{\sigma _{\tilde{N}}} (\langle \mathcal {V}''_{gd}(cb), cb, \nu _n+ 1 \rangle ). \end{aligned}$$

Upon receiving $va$, $\tilde{N}$ decrypts it and verifies it by using,

11 $$\begin{aligned} t= {\left\{ \begin{array}{ll} \text {True,} & \text{ If } \ \Phi _{\tilde{N}} = \mathcal {Q}'' \wedge qa(\nu _n+ 1) > \nu _n, \\ \text {False,} & \text {Otherwise}. \end{array}\right. } \end{aligned}$$

If $t$ is valid, the collaborator $\tilde{N}$ is ready to participate in the model training. After establishing the secure connection with all $\tilde{N}$, $\tilde{Q}$ constructs a collaborator list,

12 $$\begin{aligned} \bar{N} = \bigcup (\tilde{N}), \forall N\in [0, N], \end{aligned}$$

where $N$ denotes the total number of collaborators. For each training episode, $\tilde{Q}$ selects an active participant from $\bar{N}$ based on,

13 $$\begin{aligned} vk= \max (qd\times N, 1), \end{aligned}$$

where $qd$ represents the dropout ratio of $\bar{N}$. The final contributor list is determined as,

14 $$\begin{aligned} \bar{vk} = \bigcup (\tilde{N_n}), \forall n\in [0, vk]. \end{aligned}$$

Once the contributors are finalized, $\tilde{Q}$ generates a temporary secret key $\Phi$,

15 $$\begin{aligned} \Phi = \mathcal {B} ((\bar{vk}, \nu , \nu \times 982451653)) | \mathcal {B} : \{0,1\}^* \mapsto \{0,1\}^{bk}. \end{aligned}$$

This key is transmitted securely to all $\tilde{N}$ using their public keys,

16 $$\begin{aligned} va_n= \mathcal {V}_{\sigma _{\tilde{N}}} (\langle \Phi , \nu _n+ 1 \rangle ), \forall n\in [0, |\bar{vk}|]. \end{aligned}$$

The secret key $\Phi$ enables a lightweight and secure computing environment for training. Additionally, $\tilde{Q}$ periodically updates $\Phi$ to maintain security.

### Collaborative training

The server $\tilde{Q}$ initiates training by sending $a$ to all collaborators $\tilde{N}$ listed in $\tilde{vk}$. Each $\tilde{N_n}$ contains a dataset $\rho _n$, where $\rho _{l,n} = \{j_{l,n}, f_{l,n}\} \mid \rho _{l,n} \in \rho _n, j_{l,n} \in \mathbb {E}^q, f_{l,n} \in \mathbb {E}$. Here, $\rho _{l,n}$ represents the $l$-th sample of $\rho _n$. Since the proposed approach involves heterogeneous entities with distinct feature spaces, sharing a generic model with a fixed input count can be challenging. The proposed approach is based on an information gain ratio (IGR) for feature selection, where each client selects the optimal $i$ feature from their individual feature space. First, the entropy of the clients’ dataset is calculated as,

17 $$\begin{aligned} \mathcal {V}(\rho _n) = -\sum _{m\in M} d_m\log _2(d_m). \end{aligned}$$

Here, $\mathcal {V}(.)$ represents the entropy calculation function, $m$ signifies the number of labels within the dataset $\rho _n$, and $d$ denotes the probability of label $m$ in $\rho _n$. Next, the information gain is computed for each feature $U$, given by,

18 $$\begin{aligned} W(\rho _n| U_p) = \mathcal {V}(\rho _n) - \sum _{\forall t\in [0, T_{U_p}]} \frac{|\rho ^t_n|}{|\rho _n|} \mathcal {V}(\rho ^t_n), \forall p\in [0, P]. \end{aligned}$$

Here, $U_p$ denotes the *p*-th feature, $P$ signifies the total number of features, and $T_{U_p}$ represents the unique values in feature $U_p$. Subsequently, the intrinsic information of each $U_p$ is calculated by using,

19 $$\begin{aligned} W' (U_p) = -\sum _{\forall t\in [0, T_{U_p}]} \frac{|\rho ^t_n|}{|\rho _n|} \log _2 \left( \frac{|\rho ^t_n|}{|\rho _n|} \right) , \forall p\in [0, P]. \end{aligned}$$

Furthermore, IGR is calculated for each $U_p$, given by,

20 $$\begin{aligned} \Gamma (\rho _n| U_p) = \frac{W(\rho _n| U_p)}{W'(U_p)}, \forall p\in [0, P]. \end{aligned}$$

Following this, the top $i$ features are selected based on their IGR values. Prior to data preparation for tasks, each $\tilde{N_n}$ scales its data using a standardization technique. Then, $\tilde{N}$ prepares $\rho _n$ into multiple tasks $D$ based on n-shot k-ways, with $s$ samples and $b$ classes. Each $D$ contains a support set $\rho ^g_n$ and query set $\rho ^r_n$, given by,

21 $$\begin{aligned} D_{\rho _n, h} = \bigcup _{\forall l\in [0,b]} \bigcup _{\forall w\in [0,s]} \{ \rho ^g_{n,l,w}, \rho ^r_{n,l,w} \}, \forall h\in [0, H_n], \end{aligned}$$

and $\rho ^g_{n,l,w} \cap \rho ^r_{n,l,w} = \emptyset$. Here, $H_n$ denotes the number of tasks assigned to the $n$-th collaborator. The term $\rho ^g_{n,l,w}$ refers to the $w$-th sample from the $l$-th class in $\rho ^g_n$, while $\rho ^r_{n,l,w}$ refers to the $w$-th sample from the $l$-th class in $\rho ^r_n$. The objective is to minimize the loss function of the consolidated global model with parameters $\phi$ where $\phi \in \{x, u\}$, so that it can adapt heterogeneous data more quickly. The overall objective function can be formulated as,

22 $$\begin{aligned} \min _{\phi } \frac{1}{|\tilde{vk}|} \sum _{n\in [0,|\tilde{vk}|]} \mathcal {K}_n(\phi - \epsilon \nabla _{\phi } \mathcal {K}_n(\phi )). \end{aligned}$$

Here, $\mathcal {K}_n(.)$ represents the loss function for client $n$. This paper considers meta-learning with implicit gradients (iMAML) in the proposed approach, which employs a second-order optimization method, relying solely on the solution to the inner-level optimization. The objective of iMAML is to acquire meta-parameters that result in effective task-specific parameters following adaptation, given by,

23 $$\begin{aligned} \underbrace{\phi ^*_n:= \arg \min _{\phi _n} \mathcal {O}(\phi _n),}_{\text {outer-level}} \quad \mathcal {O}(\phi _n) = \frac{1}{H_n} \sum _{l=0}^{H_n-1} \mathcal {K}_n\left( \underbrace{\mathcal {C}(\phi _n, \rho ^g_{n,l})}_{\text {inner-level}}, \rho ^r_{n,l} \right) . \end{aligned}$$

Here, $\phi ^*_n$ is the best-learned parameter, $\mathcal {O}$ is the mean of validation loss functions, and $\mathcal {C}(.)$ is the training algorithm function. Notation $\mathcal {C}(\phi _n, \rho ^g)$ involves initializing at $\phi _n$ and applying gradient descent iteratively, once or multiple times. For one step of gradient descent, we have,

24 $$\begin{aligned} \phi '_{n,l} = \mathcal {C}(\phi _n, \rho ^g_{n,l}) = \phi _n- \epsilon \nabla _{\phi _n} \mathcal {K}_n(\phi _n, \rho ^g_{n,l}), \end{aligned}$$

where $\phi '$ is task-specific parameters. To address the problem of vanishing gradients, a simple regularization term is incorporated, ensuring that the task-specific parameters remain close to the meta-parameters,

25 $$\begin{aligned} \mathcal {C'}(\phi _n, \rho ^g_{n,l}) = \arg \min _{\phi '_n} \mathcal {K}_n(\phi '_n, \rho ^g_{n,l}) + \frac{\Psi }{2} \Vert \phi '_n- \phi _n\Vert ^2. \end{aligned}$$

Here, $\Psi$ is the regularization coefficient controlling the strength of the regularization. To reduce the computation complexity, an implicit gradient is computed by applying the first chain rule to the base form, assisting in computing the implicit Jacobian. Upon completing the training process, each $\tilde{N_n}$ encrypts $\nabla \phi _n$ using $\Phi$ and subsequently shares the encrypted data with $\tilde{Q}$.

### Consolidation process

Upon receiving $\nabla \phi _n$, $\tilde{Q}$ decrypts the message using $\Phi$ and verifies the sender using $XF$ and $CNA$, by using the Hampel filter. Each parameter is then converted into a scalar and a list of scalar values is created as follows: $g_{nN} = flatten(\phi _n), \forall n\in [0, |\vec {k}|)$ and $g_N= \cup _{n\in [0, |\vec {k}|)} (g_{nN})$. Next, a median $\mathcal {A}(g_N)$ is determined from this list, followed by calculating a standard deviation,

26 $$\begin{aligned} gq= \frac{2\Upsilon }{\sqrt{2erf^{-1}(0.5)}}, \end{aligned}$$

where $\Upsilon = \mathcal {A}_{\forall n\in [0, |g_N|)} (|g_{nN} - \mathcal {A}(g_N)|')$. Subsequently, a list of malicious collaborators is identified as,

27 $$\begin{aligned} \vec {k}^a= \{ \cup _{n\in [0, |\vec {k}|)} (k_n, |g_{nN} - \mathcal {A}(g_N)|' > dp\times gq) \}. \end{aligned}$$

Following this, malicious entities are removed from the list of collaborators,

28 $$\begin{aligned} \vec {k}' = \vec {k} - \vec {k}^a. \end{aligned}$$

A performance check (e.g., accuracy) $\mathcal {R}(.)$ is performed on the received $\nabla \phi _n$ from $\vec {k}'$ and unmodified model updates are discarded based on the list,

29 $$\begin{aligned} \vec {k}'' = \{ \cup _{n\in [0, |\vec {k}'|)} (k_n'| \mathcal {R}(\nabla \phi _n)^d> \mathcal {R}(\nabla \phi _n)^{d-1}) \}. \end{aligned}$$

Finally, the consolidation follows the FedAvg algorithm,

30 $$\begin{aligned} \phi = \frac{1}{|\vec {k}''|} \sum _{\forall n\in [0, |\vec {k}''|)} (\phi _n). \end{aligned}$$

Afterwards, $\tilde{Q}$ encrypts $\phi$ using $\Phi$ and distributes it to all $\vec {k}''$ along with $\nu _n+ 1$. Simultaneously, $\tilde{Q}$ stores the encrypted $\phi$ within $u_{CFC} = \cup ((\phi , \vec {k}'', \cup _{\forall n\in [0, |\vec {k}'|)} (\phi _n)))$. Each $\tilde{Q}$ has $LS$, supporting $FC$. Once the connectivity is established, $\tilde{Q}$ starts offloading data to the nearest MEC server $\tilde{A}$ to update $u_{CBC}$ with the latest training data. Upon receiving data from $\tilde{Q}$, $\tilde{A}$ creates a block for appending to $u_{CBC}$ for permanent storage, placing it in a queue. In the proposed approach, the rotational consensus algorithm ($RCA$) is used to ensure efficient block production even if some validators do not participate. Each miner gets a turn to propose a block, ensuring the network continuity if one fails. Upon receiving a block proposal, the miners verify duplicates and evaluate the training’s relevance based on the historical data. The protocol automatically detects and penalizes misbehavior. Violations, like proposing out of turn, are caught through timestamp checks and proposal order. Penalties can include reputation damage, temporary suspension, slashing of stakes or rewards, and permanent removal for repeated offenses. If the validation criteria including data duplicates and training relevance are met, the miners will approve the block. If not, it is rejected and the sender is penalized, delaying future proposals. Once validated by the majority, the data is permanently stored in $un_{BC}$. The whole procedure of the proposed approach is summarized in Algorithm 1.

**Algorithm 1:**
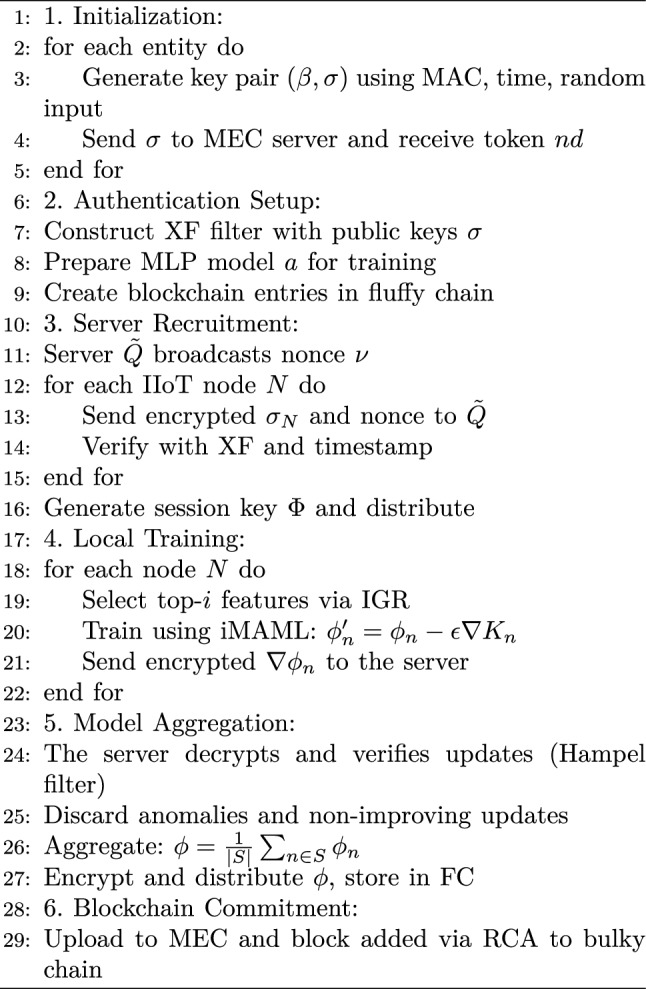
Secure Data Consolidation

### Security analysis

To evaluate the robustness of the proposed approach against the malicious manipulation, we now extend to analyze the probability of an attacker bypassing the multi-tier defense mechanism. In the considered system, malicious actors primarily attempt to interfere with the convergence of the global model $\phi$ by injecting poisoned model updates. The defense architecture consists of the following two hierarchical layers:

1. Identity authentication layer (XF authentication): This layer utilizes the XOR filter based challenge-response mechanism to verify the legitimacy of participants and prevent unauthorized devices from accessing the network.
2. Data cleaning layer (Hampel filter): During the consolidation process, the server $\tilde{Q}$ uses the Hampel filter to identify and discard statistical outliers that deviate significantly from the normal parameter distribution.

The necessary condition for a successful attack is that the malicious update should bypass both independent detection layers. In the following theorem, we provide the lower bound for the attack failure probability by combining the collision probability of identity impersonation and the statistical deviation of numerical poisoning.

#### Theorem 1

Let $N_a$ be the number of malicious entities attempting to inject poisoned gradients. The probability of attack failure, defined as $P_{fail} = 1 - P_{suc}$ (where $P_{suc}$ is the probability that a malicious update is integrated into the global model $\phi$), satisfies the following inequality,

31 $$\begin{aligned} P_{fail}&\ge 1-\epsilon _{xf}\Bigg [ \Phi \!\left( \frac{dp\cdot gq+\mathcal {A}(g_N)-\mu _p}{\sigma _p}\right) \nonumber \\&-\Phi \!\left( \frac{-dp\cdot gq+\mathcal {A}(g_N)-\mu _p}{\sigma _p}\right) \Bigg ], \end{aligned}$$

where $\epsilon _{xf}$ is the false positive rate of the XF authentication, and $\mu _{p}$ and $\sigma _{p}$ represent the mean and standard deviation of the poisoned parameter distribution, respectively. Additionally, $\Phi (\cdot )$ denotes the cumulative distribution function (CDF) of the standard normal distribution.

#### Proof

See Appendix A. $$\square$$

### Computational complexity analysis

The per-round computational complexity of the proposed approach can be decomposed into client-side and server-side components. On each participating client, the total overhead consists of (i) feature processing/selection, which scales linearly with the local sample size and raw feature dimension, and (ii) iMAML-based local training, whose dominant cost scales with the number of meta-tasks and implicit-gradient iterations, plus (iii) symmetric encryption of the transmitted update, which is linear in the model/update size. Overall, the client-side cost is dominated by the iMAML term and can be summarized as $(O!\big (P S_n + H_n(G+T_{cg})W\big ))$, with an additional linear encryption term (*O*(*W*)).

On the server, the authentication and timestamp checking scale linearly with the number of received client updates, while robust aggregation and FedAvg aggregation both scale linearly with the product of the number of participating clients and the model size, with a sorting-based implementation adding a logarithmic factor. For the blockchain layer, appending to the lightweight chain is essentially constant-time bookkeeping plus linear serialization/encryption, whereas committing to the bulky chain via RCA incurs block validation overhead upper bounded by $(O(M(B+1)))$ and typically (*O*(*M*)) communication. Combining these components yields the per-round end-to-end complexity,

32 $$\begin{aligned} \mathcal {O}\Bigg (\sum _{n=1}^{v_k}\Big (P S_n + H_n(G+T_{\textrm{cg}})W\Big )\;+\; v_k W \;+\; M(B+1)\Bigg ), \end{aligned}$$

where the dominant term is the iMAML local training cost and the remaining steps are linear in model size.

## Simulations results and discussions

**Table 3:** System configurations and parameters.

Configuration/parameter	Value
Simulation platform	Python-based simulations; workstation with Intel Core i9, 32 GB RAM, NVIDIA RTX 4090
Learning rate	0.001
Local training per communication round	5 epochs
Global training process	100 epochs
Number of participating clients	[10,15,20]
Local model	Lightweight MLP with 3 hidden layers (64, 32, 16 neurons)
Activation & regularization	PReLU; dropout rate 0.3
Meta-learning	iMAML; regularization coefficient 0.1
Dataset	SWaT
Blockchain validation	[5,10,15,25,30]miners
Block size	0–3000 bytes
Dual-chain & consensus	Fluffy chain+ bulky chain; rotational consensus
Latency threshold $\tau _{th}$	1 s

To evaluate the performance of the proposed approach, we conduct some Python-based simulations on a workstation equipped with an Intel Core i9 processor, 32 GB RAM, and an NVIDIA RTX 4090 GPU. If not specified, the learning rate is set to 0.001, the number of local training epochs per communication round is fixed at 5, and the global training process consists of 100 epochs. We vary the number of participating clients among 10, 15, and 20 to analyze the system scalability and the impact of data heterogeneity. Moreover, each client trains a lightweight multilayer perceptron model with three hidden layers of 64, 32, and 16 neurons, respectively, using the PReLU activation function and a dropout rate of 0.3 to mitigate overfitting. The meta-learning process is implemented using the implicit model-agnostic meta-learning algorithm with a regularization coefficient of 0.1.

To better emulate practical industrial IoT and enterprise IT scenarios, we adopt the Secure Water Treatment (SWaT) dataset in our simulations^37^, which represents process-control-oriented enterprise sites with high-frequency telemetry. This dataset is collected from the iTrust testbed and consists of multivariate sensor and actuator tags recorded from a six-stage water treatment plant under both normal and attack scenarios. In parallel, we simulate the blockchain-based validation layer by setting the number of miners to 5, 10, 15, and 20 to evaluate the validation latency under different network sizes, while the block size is varied from 0 to 3000 bytes of training history. The proposed dual-chain structure incorporates a lightweight fluffy chain for temporary validation and a bulky chain for permanent archival, operating under a rotational consensus algorithm, and the latency threshold $\tau _{th}$ of federated learning is set to 1s to capture the time-sensitive constraint in practical deployment. The main system configurations and parameter settings are summarized in Table 3.

**Fig. 2:**
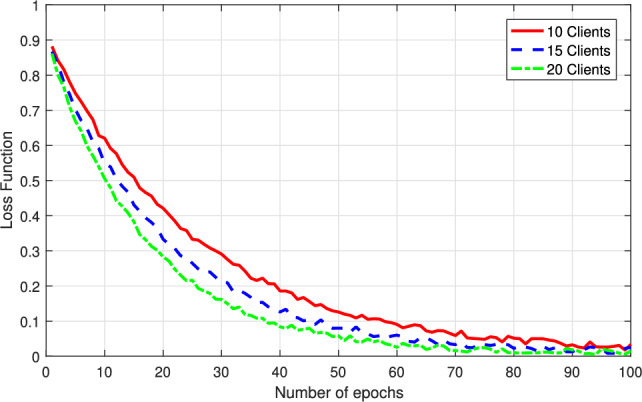
Loss function of the proposed approach versus the number of epoches.

Figure 2 depicts the variation of the loss function with respect to the number of training epochs for the proposed data aggregation approach, where $\tau =1s$ and there are 10, 15, and 20 clients in the federated learning. We can observe from Fig. 2 that for each number of clients, the loss function of the proposed approach becomes smaller as the training progresses, confirming the model’s convergence behavior and stability. Initially, the loss is relatively high for all client numbers, reflecting random weight initialization and early-stage model adjustment. As the number of epochs increases, the loss decreases rapidly during the first 20–30 epochs and then gradually stabilizes, indicating that the model is effectively learning generalizable representations even in heterogeneous data settings. Among the three configurations, the proposed approach with 10 clients achieves the fastest convergence with the lowest final loss, suggesting that fewer clients with more homogeneous local data lead to faster optimization. In contrast, the proposed approach with 15 clients exhibits slightly slower convergence but maintains a stable trajectory, while that with 20 clients shows the slowest loss reduction due to greater data heterogeneity and increased communication complexity. Nonetheless, all configurations converge toward low and stable loss values by the 80th to 100th epoch, indicating the robustness and scalability of the proposed meta-learning approach in managing diverse enterprise datasets across decentralized nodes.

**Fig. 3:**
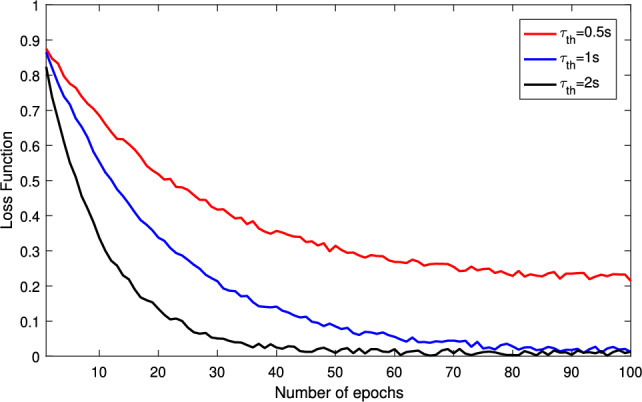
Impact of latency threshold on the training convergence of the proposed approach.

Fig. 3 illustrates the impact of the latency threshold $\tau _{\text {th}}$ on the training convergence of the proposed federated meta-learning approach, where the number of epochs varies from 0 to 100 and there are 15 clients. We consider three kinds of latency threshold constraint in the federated learning, including $\tau _{\text {th}}=0.5$s, $\tau _{\text {th}}=1$s, and $\tau _{\text {th}}=2$s. From this figure, we can find that for all three thresholds, the loss function of the proposed approach starts from a relatively high value and then decreases as the number of epochs increases, confirming that the learning process is stable and convergent under heterogeneous enterprise data. However, the curves with a larger latency threshold tend to converge faster and reach lower final loss values, while the strict latency constraint with $\tau _{\text {th}}=0.5$s exhibits the slowest decay and the highest convergence, indicating that an aggressive latency constraint forces the server to discard many delayed updates and thus harm the global model’s ability to generalize across diverse clients. This figure therefore reveals a fundamental tradeoff between the responsiveness and statistical efficiency: relaxing the latency threshold improves the convergence quality by leveraging more client updates, but beyond a certain point the marginal gain diminishes.

**Fig. 4:**
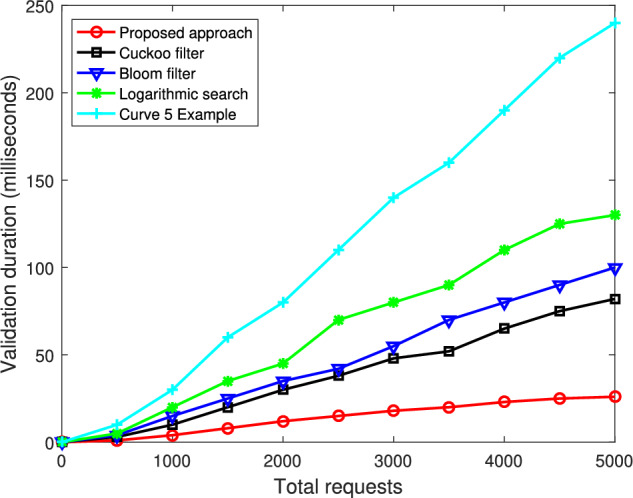
Validation duration comparison versus the total requests.

Figure 4 demonstrates the validation duration of the proposed approach versus the total requests ranging from 0 to 5000, where four competing approaches of exhaustive search, logarithmic search, bloom filter, cuckoo filter are considered for comparison. We can find from Fig. 4 that as the total number of requests increases, the validation duration of the competing approaches grows steeply, exposing the limitations under heavy workloads. Specifically, the exhaustive search suffers from extreme inefficiency, reaching nearly 250 ms at 5000 requests, while the logarithmic search remains around 180–200ms. Even the more advanced bloom filter and cuckoo filter approaches, which optimize validation through probabilistic data structures, still show significant increases in the validation delay, approximately 150 ms and 100 ms, respectively, at high request volumes. In sharp contrast, the proposed approach maintains a consistently low validation duration below 50 ms across the entire range, demonstrating the exceptional scalability and computational stability. This performance gain, roughly five times faster than exhaustive search, four times faster than logarithmic search, and twice as fast as cuckoo filter, stems from the proposed approach’s integration of federated meta-learning with a dual blockchain structure. The meta-learning mechanism enables a rapid model adaptation and efficient aggregation across heterogeneous enterprise nodes, while the lightweight blockchain layer optimizes the transaction validation and minimizes the redundant computation. Furthermore, the aggregation reduces the communication bottleneck, ensuring a high throughput even under large-scale deployments.

**Fig. 5:**
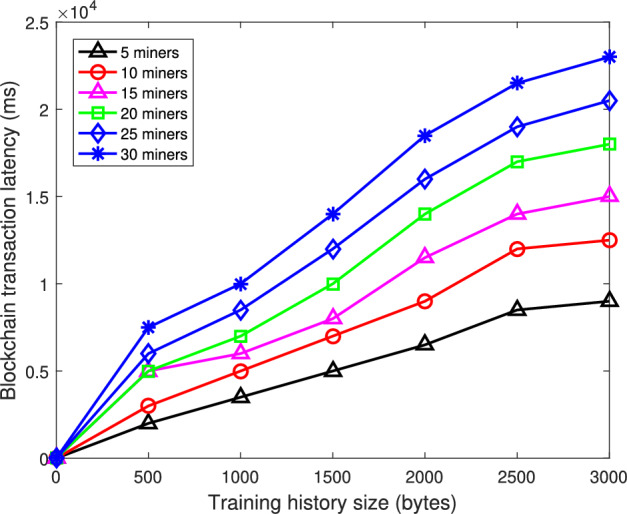
Transaction validation latency of the proposed approach in the blockchain with the training history size.

Figure 5 presents the blockchain transaction validation latency versus the training history size under different miner configurations, where there are 15 clients in the federated learning and the number of miners varies from 5 to 30. From this figure, we can see that as the training history size increases from 0 to 3000 bytes, the blockchain transaction latency of the proposed approach increases for different values of number of miners. Specifically, the blockchain transaction with 5 miners achieves the lowest latency, starting below 2000 ms and increasing only gradually to around 6000 ms at 3000 bytes of training history. In contrast, the blockchain transaction with 10 and 15 miners shows a moderately higher latency, rising to approximately 8000 ms and 12,000 ms, respectively, while the blockchain transaction with 30-miner configuration performs the worst, with latency exceeding 23,000 ms at the same training size. These results indicate that the proposed approach scales efficiently with the historical data and operates more effectively with a smaller number of miners, significantly reducing the validation delay compared to traditional methods which suffer from consensus overhead and communication congestion. The consistent low-latency performance can be attributed to the dual-chain design, where the lightweight fluffy chain handles fast temporary validation while the bulky chain manages secure permanent storage, combined with the rotational consensus algorithm, which minimizes the redundant verification and ensures efficient block production even when some miners are inactive. Furthermore, the integration of federated meta-learning optimizes the update relevance and reduces unnecessary blockchain interactions, thereby maintaining a high throughput under increasing training history loads.

**Fig. 6:**
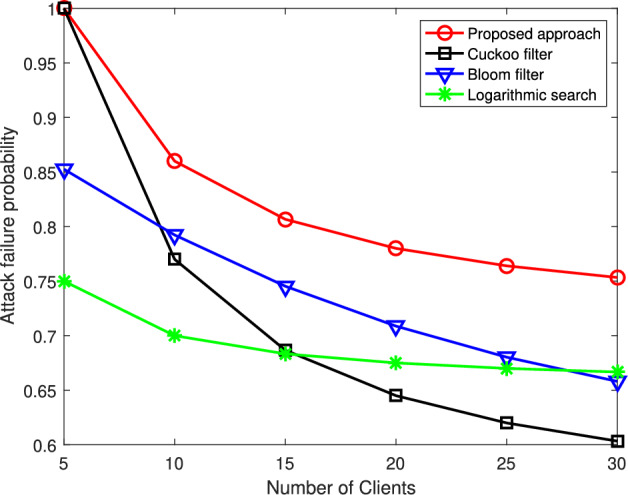
Attack failure probability versus the number of clients.

Figure 6 shows the attack failure probability with the varying number of clients of several approaches, where the number of clients varies from 5 to 30. From the results in this figure, we can find that for all approaches, the attack failure probability increases with a larger number of clients, indicating that the system becomes more vulnerable. Moreover, the proposed approach outperforms the several competing ones, for each number of client. For instance, when the number of clients is 20, the proposed approach achieves the attack failure probability of 0.78, outperforming the cuckoo filter of 0.64, bloom filter of 0.70, logarithmic search of 0.68. Even at 30 clients, the proposed approach maintains a security advantage over the competing ones, including the bloom filter and logarithmic search, thereby validating the effectiveness of its integrated XF authentication and Hampel filter-based defense mechanism in mitigating the model poisoning attacks in heterogeneous federated environments.

## Conclusions

This paper proposed a secure and scalable data aggregation framework for heterogeneous enterprise data by integrating federated meta-learning with model collection. The proposed approach addressed the challenges of data privacy, statistical heterogeneity, and limited communication bandwidth of the enterprise data. Specifically, local models were trained independently on edge devices and subsequently aggregated by the server using meta-learning to improve generalization over non-IID enterprise data. Then, a consortium blockchain with a dual-chain structure was employed, including the lightweight “fluffy chains” for transient storage and heavyweight “bulky chains” for permanent, immutable records. The proposed approach further incorporated multiple security mechanisms, including XF-based mutual authentication, timestamp-based counters for replay attack prevention, asymmetric encryption for secure key exchange, and a Hampel filter to detect and mitigate model poisoning. Simulation results on SWaT dataset were finally provided to demonstrate that the proposed approach consistently maintained a lower validation latency over competing ones, and meanwhile significantly enhanced the system security by increasing the attack failure probability by up to 15%.

Despite the encouraging results, the proposed approach may still have some practical limitations. First, integrating blockchain-based validation introduces additional computation, storage, and communication overhead, which may increase the end-to-end latency under large-scale participation or constrained edge resources. Second, the current design is evaluated under a specific set of models and datasets, and its generalization to broader heterogeneous enterprise deployments (e.g., varying data distributions, device capabilities, and network conditions) requires further verification. Future work will therefore focus on designing lightweight and adaptive validation mechanisms (e.g., dynamic miner selection, adjustable block/history management, and latency-aware scheduling) to reduce the overhead while maintaining trustworthiness, and on conducting prototype-level experiments in representative industrial edge environments with more diverse tasks and datasets to systematically assess scalability, robustness, and operational cost.

## Data Availability

The datasets used and/or analysed during the current study available from the corresponding author on reasonable request.

## Appendix A

In this appendix, we provide the proof of Theorem 1. For a malicious update to be effective, an attacker should satisfy two independent random events: successful authentication (Event *A*) and bypassing the filter (Event *B*). For these two events, we consider the associated attack probability, detailed as follows,

(1) Authentication bypass probability (Event *A*): In this event, the attacker cannot obtain a legitimate private key $\beta$ or the corresponding authentication token $nd$. The probability of bypassing the system via a collision is limited by the capacity factor of the XF structure, given as $P(A) = \epsilon _{xf}$. Due to the design where $dk= 1.23 \times | {vd} | + 32$, this probability is kept at a negligible level.

(2) Filter bypass probability (Event *B*): In this event, even if authenticated, the malicious update $g_{nN}$ should fall within the acceptance interval of the Hampel filter, such that $|g_{nN} - \mathcal {A}(g_N)| \le dp\times gq$. Assuming that the poisoned parameters follow a normal distribution $X_p \sim \mathcal {N}(\mu _p, \sigma _p^2)$, the probability that an item falls in the interval $[\mathcal {A}(g_N) - dpgq, \mathcal {A}(g_N) + dpgq]$ is calculated by applying standard normalization,

A.1 $$\begin{aligned} P(B) = \Phi \left( \frac{\mathcal {A}(g_N) + dpgq- \mu _{p}}{\sigma _{p}} \right) - \Phi \left( \frac{\mathcal {A}(g_N) - dpgq- \mu _{p}}{\sigma _{p}} \right) . \end{aligned}$$

From *P*(*A*) and *P*(*B*), we can compute the attack success probability as $P_{suc} = P(A) \cdot P(B)$, as the identity authentication and parameter injection are sequential and independent events. Thus, the total failure probability $P_{fail} = 1 - P_{suc}$ yields the inequality defined in Theorem 1. This result indicates that as the poisoning deviation $|\mu _p - \mathcal {A}(g_N)|$ increases, *P*(*B*) tends to zero, and the attack failure rate $P_{fail}$ approaches to 1 exponentially.
